# Supplementary material for: Rapid turnover of life-cycle-related genes in the brown algae
Source: Genome Biol. 2019 Feb 14;20:35. doi: 10.1186/s13059-019-1630-6 (PMC6374913; doi:10.1186/s13059-019-1630-6)
Supplement: Supplementary file 3 — Protein alignments, corresponding Gblocks html file and CDS sequences for the lineage conserved gametophyte-biased genes. (ZIP 2109 kb) [file 13059_2019_1630_MOESM3_ESM.zip › Con_GA_alignments/Ec-25_000580.aa_ali.fasta-gb.htm]

Ec-25\_000580.aa.fasta.trans.aa\_ali.fasta


## Gblocks 0.91b Results

Processed file: **Ec-25\_000580.aa.fasta.trans.aa\_ali.fasta**  
Number of sequences: **2**  
Alignment assumed to be: **Protein**  
New number of positions: **161** (selected positions are underlined in blue)

```
                         10        20        30        40        50        60
                 =========+=========+=========+=========+=========+=========+
Ec-25_000580     MACEDREAAVAQEKPATTPLSPEEDESVDDERKATSEPEAGTEGTSALAAVAKPEYSIDQ
MSTRG.36173.1    ------------------------------------------------------------
                                                                             


                         70        80        90       100       110       120
                 =========+=========+=========+=========+=========+=========+
Ec-25_000580     TYTTALLRGRHPRSFRNERGIYQYVKDSNKRANMGQKGHAQRHNRPALADPNSLLARSSG
MSTRG.36173.1    ------------------------------------------------------------
                                                                             


                        130       140       150       160       170       180
                 =========+=========+=========+=========+=========+=========+
Ec-25_000580     GGSGDLDRSLRSQTTVGVHFFASSSSPQDDNVHERSNNNNNNNEDDNRRGHQLPSSRSSR
MSTRG.36173.1    ------------------------------------------------------------
                                                                             


                        190       200       210       220       230       240
                 =========+=========+=========+=========+=========+=========+
Ec-25_000580     SPRPATATTSSASLERGGSCQHVATGTGEQPLQQGNSQNSSREASVLYVGGARARSAGGG
MSTRG.36173.1    ------------------------------------------------------------
                                                                             


                        250       260       270       280       290       300
                 =========+=========+=========+=========+=========+=========+
Ec-25_000580     GGGGGSRQRQPQPVAPSGGGGGEQLQQSVAPSEPNTAGKDGGGGGGSRRRPRKGLLASRG
MSTRG.36173.1    ------------------------------------------------------------
                                                                             


                        310       320       330       340       350       360
                 =========+=========+=========+=========+=========+=========+
Ec-25_000580     AFPSKGPPAPLGRGGSSLLGGYVIQQGGSFRGGGGEASGLFPGADGLAGGGRGTSGHGGQ
MSTRG.36173.1    ------------------------------------------------------------
                                                                             


                        370       380       390       400       410       420
                 =========+=========+=========+=========+=========+=========+
Ec-25_000580     QASWGRAGDRPMSLHQKFNAMVGIQRESSSREEEGTQVLDSLNHDPSLRLHYGLHVTFQA
MSTRG.36173.1    ------------------------------------------------------------
                                                                             


                        430       440       450       460       470       480
                 =========+=========+=========+=========+=========+=========+
Ec-25_000580     VQGENGYFMSLDMETGGVSMIPLHELQKNNQVTFKMIDLEDPESYRSIFYGKPVWLQIVP
MSTRG.36173.1    ------------------------------------------------------------
                                                                             


                        490       500       510       520       530       540
                 =========+=========+=========+=========+=========+=========+
Ec-25_000580     GPGEPTWRDGSVLGARVHGPGMLPTVVIDPNDAAAASSMAHQTAHSRPGTSESTPGPGTR
MSTRG.36173.1    ------------------------------------------------------------
                                                                             


                        550       560       570       580       590       600
                 =========+=========+=========+=========+=========+=========+
Ec-25_000580     RPVSTHGASSGRGNTGWNARDSPGGGGGWAESSFLPGGGGGGGGGGGGEVVGAGRVSSAV
MSTRG.36173.1    ------------------------------------------------------------
                                                                             


                        610       620       630       640       650       660
                 =========+=========+=========+=========+=========+=========+
Ec-25_000580     GGGNTDANRPVGVPYPFKAAGSKHGGAGGTANETLLRAMNKSAIVNCRWTVRPVRSDGQS
MSTRG.36173.1    ------------------------------------------------------------
                                                                             


                        670       680       690       700       710       720
                 =========+=========+=========+=========+=========+=========+
Ec-25_000580     LLGKDGKEVSNLDYVYLEQDFFYMTRVSENASVVLKALSHNNRSAGSNGGGGGGSASGKR
MSTRG.36173.1    ------------------------------------------------------------
                                                                             


                        730       740       750       760       770       780
                 =========+=========+=========+=========+=========+=========+
Ec-25_000580     GTAQKHHPVGRRGVFKLQVADADELRPGEERSAALMRKAKRGLKRSEARRSGHRTYVHKQ
MSTRG.36173.1    -------------------------------SAALMRKAKRGLKRSEARRSGHRTYVHRQ
                                                #############################


                        790       800       810       820       830       840
                 =========+=========+=========+=========+=========+=========+
Ec-25_000580     RPQDRLTSGAAFPREIRVRTTHFVSESASRAISSASHSPHRGG--GAGGGEEGGVVGYSR
MSTRG.36173.1    RPEERLTSGAAFPREIRVRTTHFASESESRAISSASQSPPHGAIIGSGGSGKARAVGHSQ
                 ##########################################   ###############


                        850       860       870       880       890       900
                 =========+=========+=========+=========+=========+=========+
Ec-25_000580     SG-VSLGG-------SWDGFGDGGGGSAWLS---------AGSWGTDVLQAMTDFARAGA
MSTRG.36173.1    GGGTGAGGGGYGGEGDWDCYGNGGG-SAWLTSSSAASLFHTGSWGSKLLQAMTDAARAPP
                 ##                                       ###################


                        910       920       930       940       950       960
                 =========+=========+=========+=========+=========+=========+
Ec-25_000580     ERGVTASHARVLREWKQRWGPLGTIWSQADDHSSRGTTGDSSDSNGEGEDGGGDEGHGWS
MSTRG.36173.1    GCGVTASHARVLREWRQRWGHLSVHSSQADDSSSLGSATGHDD-----------------
                 ###########################################                 


                        970       980       990      1000      1010      1020
                 =========+=========+=========+=========+=========+=========+
Ec-25_000580     LVSPMCSRCRYLTAHPLTPPPPAGTIGTCSSRSLSSSASMTTTTTTDEAEVTLGRYCLCT
MSTRG.36173.1    ----------------------------YSSGNLSG------------------------
                                                                             


                       1030      1040      1050      1060      1070      1080
                 =========+=========+=========+=========+=========+=========+
Ec-25_000580     VNREIQTEVLQGDASGGHARAATAEPGAALSVGTEAGHEPSVSSPTAEGGAPSGSPAASR
MSTRG.36173.1    --------IDPGDADHGHR-----------------------------------------
                                                                             


                       1090      1100      1110      1120      1130      1140
                 =========+=========+=========+=========+=========+=========+
Ec-25_000580     MSTPAGGAASGMNASEMGGELALEGAGRPVGGASERNTSLGGGGGCGGGLSRMPSSNTLA
MSTRG.36173.1    ----------------------------------------GRGGGSG-------------
                                                                             


                       1150      1160      1170      1180      1190      1200
                 =========+=========+=========+=========+=========+=========+
Ec-25_000580     LAAGGFAAGGAGGAIGGLKRARDRRADRRKKKDPFEALEERLLDGFSLESITKRLQQQDE
MSTRG.36173.1    --------------------------------------------------------DEDD
                                                                             


                       1210      1220      1230      1240      1250      1260
                 =========+=========+=========+=========+=========+=========+
Ec-25_000580     TLQKGVRHVERMESRRHEPGRRSNEEHQRYLMVQKKRFEAVVSDSIAESITGSSLSAGDE
MSTRG.36173.1    ---RGGVHVSPMCPRCX-------------------------------------------
                     ###########                                             


                       1270      1280      1290
                 =========+=========+=========+=
Ec-25_000580     RGSEDAPMVDDARGANVVAASKKVASDENAD
MSTRG.36173.1    -------------------------------
```

```
Parameters used
Minimum Number Of Sequences For A Conserved Position: 2
Minimum Number Of Sequences For A Flanking Position: 2
Maximum Number Of Contiguous Nonconserved Positions: 8
Minimum Length Of A Block: 10
Allowed Gap Positions: None
Use Similarity Matrices: Yes
```

```
Flank positions of the 4 selected block(s)
Flanks: [752  822]  [826  842]  [882  943]  [1205  1215]  

New number of positions in Ec-25_000580.aa.fasta.trans.aa_ali.fasta-gb:  161  (12% of the original 1291 positions)
```
